# Deciphering the Molecular Interaction Between the Adhesion G Protein-Coupled Receptor ADGRV1 and its PDZ-Containing Regulator PDZD7

**DOI:** 10.3389/fmolb.2022.923740

**Published:** 2022-06-28

**Authors:** Baptiste Colcombet-Cazenave, Florence Cordier, Yanlei Zhu, Guillaume Bouvier, Eleni Litsardaki, Louise Laserre, Marie S. Prevost, Bertrand Raynal, Célia Caillet-Saguy, Nicolas Wolff

**Affiliations:** ^1^ Channel Receptors Unit, UMR CNRS 3571, Institut Pasteur, Université de Paris, Paris, France; ^2^ Complexité du Vivant, Sorbonne Université, Paris, France; ^3^ Structural Bioinformatics Unit, UMR CNRS 3528, Institut Pasteur, Université de Paris, Paris, France; ^4^ Biological NMR and HDX-MS Technological Platform, UMR CNRS 3528, Institut Pasteur, Université de Paris, Paris, France; ^5^ Molecular Biophysics Platform-C2RT, UMR CNRS 3528, Institut Pasteur, Université de Paris, Paris, France

**Keywords:** deafness, G protein-coupled receptor, PDZ-containing protein, ADGRV1, PDZD7, PDZ tandem

## Abstract

Hearing relies on the transduction of sound-evoked vibrations into electrical signals, occurring in the stereocilia bundle of inner ear hair cells. The G protein-coupled receptor (GPCR) ADGRV1 and the multi-PDZ protein PDZD7 play a critical role in the formation and function of stereocilia through their scaffolding and signaling properties. During hair cell development, the GPCR activity of ADGRV1 is specifically inhibited by PDZD7 through an unknown mechanism. Here, we describe the key interactions mediated by the two N-terminal PDZ domains of PDZD7 and the cytoplasmic domain of ADGRV1. Both PDZ domains can bind to the C-terminal PDZ binding motif (PBM) of ADGRV1 with the critical contribution of atypical C-terminal β extensions. The two PDZ domains form a supramodule in solution, stabilized upon PBM binding. Interestingly, we showed that the stability and binding properties of the PDZ tandem are affected by two deafness-causing mutations located in the binding grooves of PDZD7 PDZ domains.

## Introduction

Cochlear hair cells are the primary receptors for the detection of sound-induced vibrations. Each cell possesses a bundle of actin-based stereocilia organized in rows of increasing height. The shape of the bundle is critical for the cohesive deflection of the stereocilia necessary for the cell function. Morphogenesis of the hair bundle is regulated during cochlear development, but the underlying signaling pathways remain elusive. The ankle link protein complex, transiently expressed at the base of the stereocilia, is the main candidate for regulating bundle morphogenesis, with pathogenic variants of its composing proteins leading to defects in stereocilia organization and impaired auditory function. The genes encoding these proteins are affected by mutations responsible for hereditary sensory diseases, notably the Usher syndrome type 2 (Usher2) that combined congenital deafness and progressive blindness. The protein complex is thus referred to as Usher2 complex. It encompasses two transmembrane proteins, usherin and ADGRV1, exposing very large N-terminal extracellular regions (5,011 and 5,879 residues in human, respectively) thought to form fibrous links in between stereocilia. Usherin is a single-pass transmembrane protein of largely unknown function. It contains multiple laminin and fibronectin type domains that are likely promoting extracellular matrix adhesion and local scaffolding. ADGRV1 (Adhesion G-protein Receptor V1) is a seven-pass transmembrane protein of the adhesion G protein-coupled receptor (GPCR) (Bjarnadottir et al., 2007) family B (Harmar, 2001). Its extracellular region contains multiple protein-protein and calcium interaction domains, supporting potential calcium-dependent scaffolding properties. Additionally, the extracellular region of ADGRV1 contains a GAIN domain located upstream of the first transmembrane segment, comprising a G protein receptor Proteolytic Site (GPS) triggering autoproteolysis of the protein. Self-catalyzed hydrolysis of the adhesion GPCR at the GPS cleaves the large extracellular domain from the GPCR β subunit. Both fragments might remain non-covalently bound until another trigger, such as a rearrangement of the extracellular region, disrupts the interaction (Araç et al., 2012). The new N-terminal end of the protein then consists of an extracellular Stachel peptide (Demberg et al., 2017) thought to act as an agonist and constitutively activate the GPCR (Stoveken et al., 2015), leading to a decrease of cellular cAMP through the G_αi_ signaling pathway (Hu et al., 2014). The ADGRV1 cleaved β subunit possesses a 152 residues long cytoplasmic domain with a C-terminal PDZ Binding Motif (PBM) promoting the interaction with the PDZ-containing proteins whirlin and PDZD7 (Hu et al., 2014; Zou et al., 2017). These two cytoplasmic proteins from the Usher 2 complex in turn associate with various partners such as actin binding proteins (Li et al., 2017; Du et al., 2018; Zhu et al., 2020). The formation of the PBM dependent PDZD7-ADGRV1 complex downregulates the GPCR activity and restores cAMP levels in cellular models (Hu et al., 2014). However, the structural determinants mediating PDZD7-ADGRV1 complex formation remained undetermined. Furthermore, multiple pathogenic variants of PDZD7 have been identified in patients suffering from Autosomal Recessive Non-Syndromic Hearing Loss (ARNSHL) (Schneider et al., 2009; Booth et al., 2015; Vona et al., 2016; Fuster-García et al., 2018; Guan et al., 2018; Lee et al., 2019; Luo et al., 2019; Fahimi et al., 2021). Interestingly, two human pathogenic missense variants, consisting of the same amino acid substitution, are located at the same position in the binding groove of the first (G103R) and second (G228R) PDZ domains of PDZD7 (Booth et al., 2015). Here we deciphered the contribution of the N-terminal PDZ region of PDZD7 and the cytoplasmic domain of ADGRV1 in the PDZD7-ADGRV1 interaction using complementary biophysical approaches. We highlighted the role of proximal PDZ extensions that tune the affinity towards ADGRV1. The two PDZ domains and their extensions form a supramodule in solution, able to simultaneously bind ADGRV1 PBM. Finally, we characterized the effect of the two deafness associated variants on the protein stability and on complex formation, disrupting the interaction between full-length PDZD7 and ADGRV1 β subunit, underlying potential defects in hair bundle morphogenesis.

## Materials and Methods

### Protein Expression and Purification

All PDZD7 constructs are based on the murine long isoform (Uniprot entry E9Q9W7-1); PDZ1 (81-167), PDZ1ext (81-192), PDZ2 (206-292), PDZ2ext (206-313), P1P2 (81-313), N-PDZ1ext (1-188). All were expressed in fusion with a N-terminal GST tag followed by a Tobacco Etch Virus (TEV) protease site. Protein production is performed in *E. coli* BL21 DE3 in LB or M9 minimal medium supplemented with ^15^NH_4_Cl and/or ^13^C-glucose, induced with 0.2 mM Isopropyl β-D-1-thiogalactopyranoside (IPTG) and performed overnight at 18°C. Lysis is performed in 550 mM NaCl, 2 mM β-mercaptoethanol, 250 mM EDTA and complemented with anti-proteases (Roche Diagnostics) (buffer and pH as indicated for the respective experiment) using a Constant Systems Ltd. cell disruptor at 1.3 kbar. After 1 h centrifugation at 19000G, tagged proteins are purified from the supernatant by affinity chromatography using a GSTrap (Cytiva) column followed by overnight (on-column) TEV cleavage. The cleaved proteins are eluted in 300 mM NaCl, 0.5 mM tris(2-carboxyethyl)phosphine (TCEP), 250 mM EDTA complemented with anti-proteases (buffer and pH identical to the lysis step). A last step of purification is performed by size-exclusion chromatography (SEC) using a Sephacryl S-100 HP 16/60 (Cytiva).

ADGRV1 cytoplasmic domain is based on the murine long isoform (CCDS36737; 6149-6298) and is expressed in fusion with a N-terminal Histidine tag followed by a TEV protease site. Protein production is performed in *E. coli* BL21 DE3 in LB, induced with 1 mM IPTG for 3 h at 30 °C. Lysis is done in Tris HCl pH 7.5 50 mM, NaCl 300 mM, 2 mM β-mercaptoethanol and complemented with anti-proteases using a Constant Systems Ltd. cell disruptor at 1.3 kbar. After 1 h centrifugation at 19000G, the protein is purified from inclusion bodies retrieved in the insoluble fraction. Solubilization is performed using 6M guanidine hydrochloride and the resulting fraction is loaded on a HIStrap (Cytiva) column. Guanidine hydrochloride is gradually washed using Tris HCl pH 7.5 50 mM, NaCl 150 mM, 0.5 mM TCEP complemented with anti-proteases. The His-tag is removed by overnight (on-column) TEV cleavage. The cleaved protein is subsequently eluted and a last step of purification is performed by SEC using a Sephacryl S-100 HP 16/60 (Cytiva).

### Nuclear Magnetic Resonance (NMR)

All experiments were recorded at 25°C on a Bruker 800 MHz spectrometer equipped with a triple resonance ^1^H{^13^C/^15^N} cryoprobe. The ^1^H, ^15^ N and ^13^C backbone resonances assignment of PDZ1ext was carried out using 3D HNCA, HNCACB, HNCO, HNCOCA experiments (Tris HCl buffer adjusted to pH 6). The 2D ^1^H-^15^N HSQC spectra used to compare PDZ1, PDZ1ext, PDZ2 and PDZ2ext were recorded in Tris HCl pH 7.5 (50 mM). The 2D ^1^H-^15^N HSQC spectra used to compare PDZ1ext, PDZ2ext and P1P2 were recorded in Phosphate Buffer pH 6.5 (50 mM). Titration experiments of PDZ1 (WT), PDZ1ext (WT) and PDZ1ext (G103R) against ADGRV1pbm peptide were performed in Tris HCl pH 7.5 (50 mM). Averaged Chemical Shifts Perturbations (CSP) were calculated as: Δδ_avg_ = ((Δδ(^1^H))^2^+(Δδ(^15^N)*0.159)^2^)^½^. For the determination of dissociation constants (Kd) of each peptide/PDZ interaction, averaged CSP δ_avg_ was plotted as a function of the molar ratio (peptide/PDZ) for several peaks and fitted using nonlinear regression and assuming a simple complex formation model. The average and standard deviation on minimum 10 significantly shifting peaks were calculated for each complex.

### Small-Angle X-Ray Scattering (SAXS)

X-ray scattering data were collected at the SWING beamline at Soleil Synchrotron (Saclay, France). 50 μl of sample are injected on a Superdex-75 5/150 GL size exclusion column in-line with the SAXS measuring cell. PDZD7 samples are prepared in the same buffer (Phosphate Buffer 50 mM pH 6.5, NaCl 300 mM, TCEP 0.5 mM, anti-proteases). The experiment is performed at initial concentrations of 119 μM (3.1 mg/ml) for P1P2 WT and 108 μM (2.8 mg/ml) for P1P2 G103R. For complexes, 2 μl of pH-adjusted ADGRV1pbm peptide (VELRRIPIADTHL_-COOH_; 13,7 mM) are added to the 50 μl from the sample 1 hour before recording. Initial data processing was performed using sxCuBE, FOXTROT and PRIMUS. The radii of gyration were evaluated using Guinier approximation. The Dmax was determined from the distance distribution function P(r) obtained with the program GNOM.

### Analytical UltraCentrifugation (AUC)

AUC experiments were performed at 10°C using an analytical Beckman Coulter Optima ultracentrifuge equipped with a AN50-Ti rotor. The P1P2 sample was prepared at 0.4 mg/ml in Phosphate Buffer pH 6.5 (50 mM). For the complex, 10 μl of pH-adjusted ADGRV1pbm peptide (13,7 mM) were added to 300 μl of P1P2 sample. Interference profiles were analyzed with SEDFIT.

### Metadynamics

The metadynamics simulation ran for 100 ns with four replicas for each experiment. The triple-strand conformation predicted by AlphaFold was used as starting point for PDZ1ext, and a homology model (Eswar et al., 2006) based on whirlin second PDZ for PDZ2ext. The Beta Collective Variable has been applied on the β strand extension for residues 92-117 and 92-113 for PDZ1ext and PDZ2ext respectively. We used Gaussians of height 1.28 kJ/mol with a width of 0.02, adding a Gaussian every 5 ps. A lower wall bias at a β propensity of one was used to compensate for the entropically favored unfolded states. The free energy associated with each conformation was calculated (Bonomi et al., 2009).

### Fluorescence Titrations

Fluorescence titrations were performed on a Jasco V-730 spectrometer using quartz cell 109.004F-QS (Hellma Analytics) with an optical path of 10 mm and with thick side to reduce the inner filter effect. PDZ1ext and P1P2 were prepared in Phosphate Buffer pH 6.6 (50 mM) at 6 and 2 µM respectively, while PDZ2ext was prepared in Tris HCl pH 7.5 (50 mM) at 5 µM. Variations in the quantum yield of fluorescence of tryptophans from the sample were monitored upon progressive addition of buffered ADGRV1pbm peptide. After peptide addition, the sample is homogenized by in-spectrometer magnet agitation for 30 s. The sample is left to rest for an additional 60 s before measurement. Excitation is set at 295 nm and fluorescence was measured at the maximum emission wavelength (346-349 nm) of the unbound form with a 5 nm bandwidth for both. Titration curves were fitted using the following formula: y = F_p_∗p_0_+(F_x_-F_p_)∗0.5∗(R∗I_0_+(1-R)∗x + K-((R∗I_0_+(1-R)∗x + K)^2^-4∗R∗(I_0_-x)∗x)^1/2^), where p_0_ is the initial protein concentration, I_0_ the peptide stock concentration, R the p_0_/I_0_ ratio, x the volume of added peptide in µl, F_p_ the fluorescence of PDZ constructs, F_x_ the fluorescence of the complex, and K the Kd. The values of p_0_, I_0_ and R are kept constant.

### MicroScale Thermophoresis (MST)

MST titrations were performed on a Monolith (Nanotemper) spectrometer using Monolith NT. LabelFree Premium Capillaries. The PDZ2 sample was prepared at 140 μM in MES pH6 (50 mM) and mixed (1:1) with a serial dilution (½) of ADGRV1pbm peptide (13,7 mM stock). MST traces were read out at 20 s where signal to noise ratio was maximum and titration curves were fitted using the following formula: y = U+(B-U)∗(c + c_max_ + K-((c + c_max_ + K)^2^-4c∗c_max_)^1/2^/(2c_max_). U is the F_norm_ signal of the target, B the F_norm_ signal of the complex, c the ligand concentration, c_max_ the final concentration of the ligand in the assay and K the dissociation constant.

### NanoDifferential Scanning Fluorimetry (nanoDSF)

NanoDSF experiments were performed on a Prometheus (Nanotemper) spectrometer using Prometheus NT.48 Series nanoDSF Grade High Sensitivity Capillaries. All samples were prepared in MES pH6 (50 mM) at micromolar concentrations. For the complexes, ADGRV1pbm peptide was added at a final concentration of 756 μM, diluting the samples by 5%.

### Pull-Down Experiments

HeLa cells were maintained in Dulbecco’s modified Eagle’s medium (DMEM) containing 10% fetal calf serum (FCS). PDZD7 constructs correspond to the full-length murine sequence in fusion with a N-terminal strep tag and a C-terminal GFP, wild-type or carrying G103R/G228R variants, cloned in a PMT3 vector. The ADGRV1 construct corresponds to the murine β subunit of the receptor (5884-6298) cloned in a pRK5 vector. Plasmids were transfected in separate HeLa cultures using 25 μg (PDZD7) and 10 μg (ADGRV1) of DNA per 10 cm dish using TurboFect™ Transfection Reagent (ThermoFisher Scientific) at 3 μl per μg of transfected DNA. Mixes were prepared in volumes of 1 ml/plate (in DMEM), vortexed and incubated up to 30 min at room temperature, cell media were renewed before transfection. After drop by drop addition of the transfection mix, cells were maintained at 37°C for 14 h for protein expression. Cells were dissociated using Gibco™ Trypsine-EDTA (0,05%), subsequently inactivated by addition of DMEM containing 10% FCS. Cells were counted, pelleted and resuspended at similar concentrations in PBS Tween 0.01%, Dodécyl-β-D-maltoside (DDM) 0.1% containing anti-proteases (buffer). Benzonase (Sigma) was added before sonication of the cells (40 mA amplitude, 10 pulses of 12 s with 18 s intervals, in ice). An identical volume of ADGRV1 β transfected cell lysate was mixed to either PDZD7 WT, G103R or G228R transfected cell lysates. PDZ proteins have different expression levels, meaning that different amounts of sample are needed to saturate the dynabeads with the PDZ proteins. Pull down experiments have been performed several times allowing us to estimate the yield of expression of each PDZ construct for a given amount of cells. Therefore, volumes of added PDZD7 lysate were adjusted to compensate for expression disparities between WT and mutants and maintain a similar bait concentration in each mix. Lysates from non-transfected cells were added to each mix to level total cell content to ensure the same lysate concentration in the input fraction. Dynabeads™ Protein G (Invitrogen) with coupled α-GFP rabbit antibody (Invitrogen A11122) was added to each mix (equivalent to 15 μl of bead stock per condition), then incubated 1 h on wheel (4°C). The supernatant was removed (unbound fraction) and beads were washed twice with buffer containing 10% BSA and twice with buffer without BSA. A final 40 μl wash of buffer was performed to check for remaining non-specific binding (wash fraction). The bait and bound proteins were eluted in 40 μl Bolt™ LDS Sample Buffer 4X by heating (100°C, 5 min) and vortexing (15 s). Samples were loaded twice onto duplicate 4% polyacrylamide gels along with PageRuler™Plus protein ladder (ThermoFisher Scientific) and transferred to PVDF membranes. Membranes were blocked using 5% milk TBST. Target proteins were detected using mouse α-GFP (LivingColors™ JL-8, Takara) and rabbit α-ADGRV1 (custom, raised against the cytoplasmic domain) antibodies (1/2000 in 5% milk TBST), as well as secondary α-mouse and α-rabbit IgG antibodies coupled to HRP (1/2000 in 5% milk TBST). One duplicated gel was used for each primary antibody. Signal was generated using Immobilon Forte Western HRP substrate.

## Results

**FIGURE 1:**
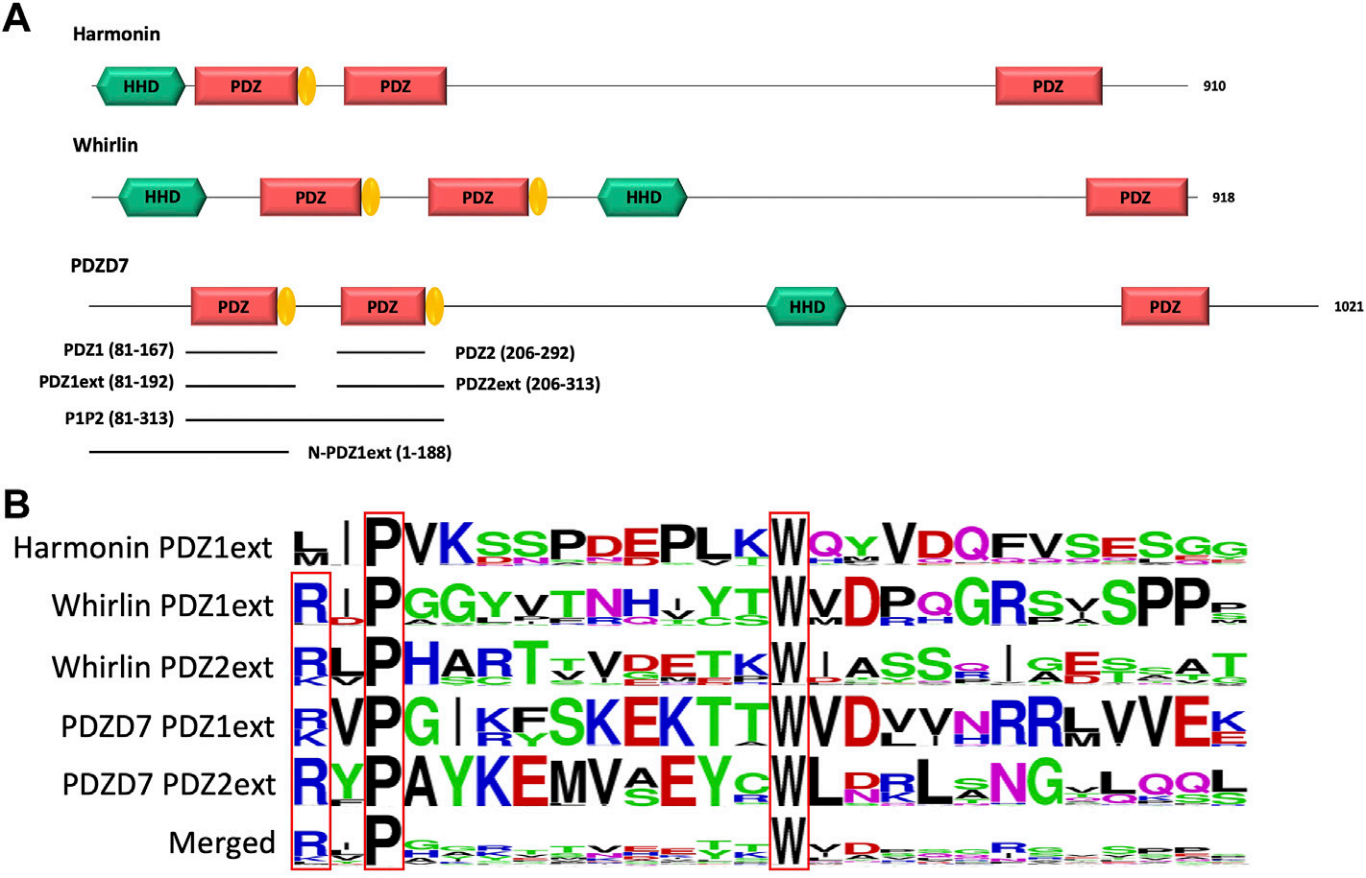
**(A)** Schematic representation of the modular organization of the three paralogous proteins harmonin, whirlin and PDZD7. PDZ domain extensions are indicated in yellow. The 6 constructs of PDZD7 used in our *in vitro* study are indicated by black lines aligned to the corresponding fragment of PDZD7. Indicated delimitations correspond to the mouse protein sequence. **(B)** Comparison of PDZ domain extension sequences from harmonin, whirlin and PDZD7. For each extension, all UniprotKB sequences of the corresponding protein were aligned. The result is displayed using a logo representation, where the height of each residue one-letter code translates to its conservation at the corresponding position in the sequence alignment (Crooks et al., 2004).

The N-terminal PDZ domains of PDZD7 paralogous proteins harmonin and whirlin possess C-terminal extensions with conserved proline and tryptophan residues (Figure 1). These folded extensions modulate the affinity of associated PDZ domains for their partners and promote the formation of domain assembly. In harmonin, this extension folds as a hairpin followed by a short α-helix mediating the formation of a heterotopic supramodule between the first PDZ and the upstream Harmonin Homology Domain (HHD) and forming direct contacts with residues upstream of the partner’s PBM (Yan et al., 2010) (PDB entry 3k1r). This supramodule displays ∼10 fold increased affinity for protocadherin-15 compared to the isolated PDZ (Michel et al., 2020). In whirlin, the first and second PDZ domains interact via their respective C-terminal extensions, structured as hairpins, promoting the formation of a homotypic supramodule that improves the binding capacity of the PDZ tandem by ∼3 fold (Delhommel et al., 2017). In PDZD7, homologous extensions with conserved P and W residues are also identified downstream the two N-terminal PDZ domains (Figure 1). We designed four constructs of the isolated N-terminal PDZ domains of mouse PDZD7 to decipher the role of the PDZ downstream sequences in the interaction with the C-terminal motif PBM of ADGRV1 (ADGRV1pbm). These constructs correspond to the first PDZ domain (PDZ1 construct; A81 to G167), the first PDZ with its C-terminal extension (PDZ1ext construct; A81 to E192), the second PDZ (PDZ2 construct; G206 to G292) and the second PDZ with its C-terminal extension (PDZ2ext construct; G206 to G313).

### Folding of the PDZ1 Extension

**FIGURE 2:**
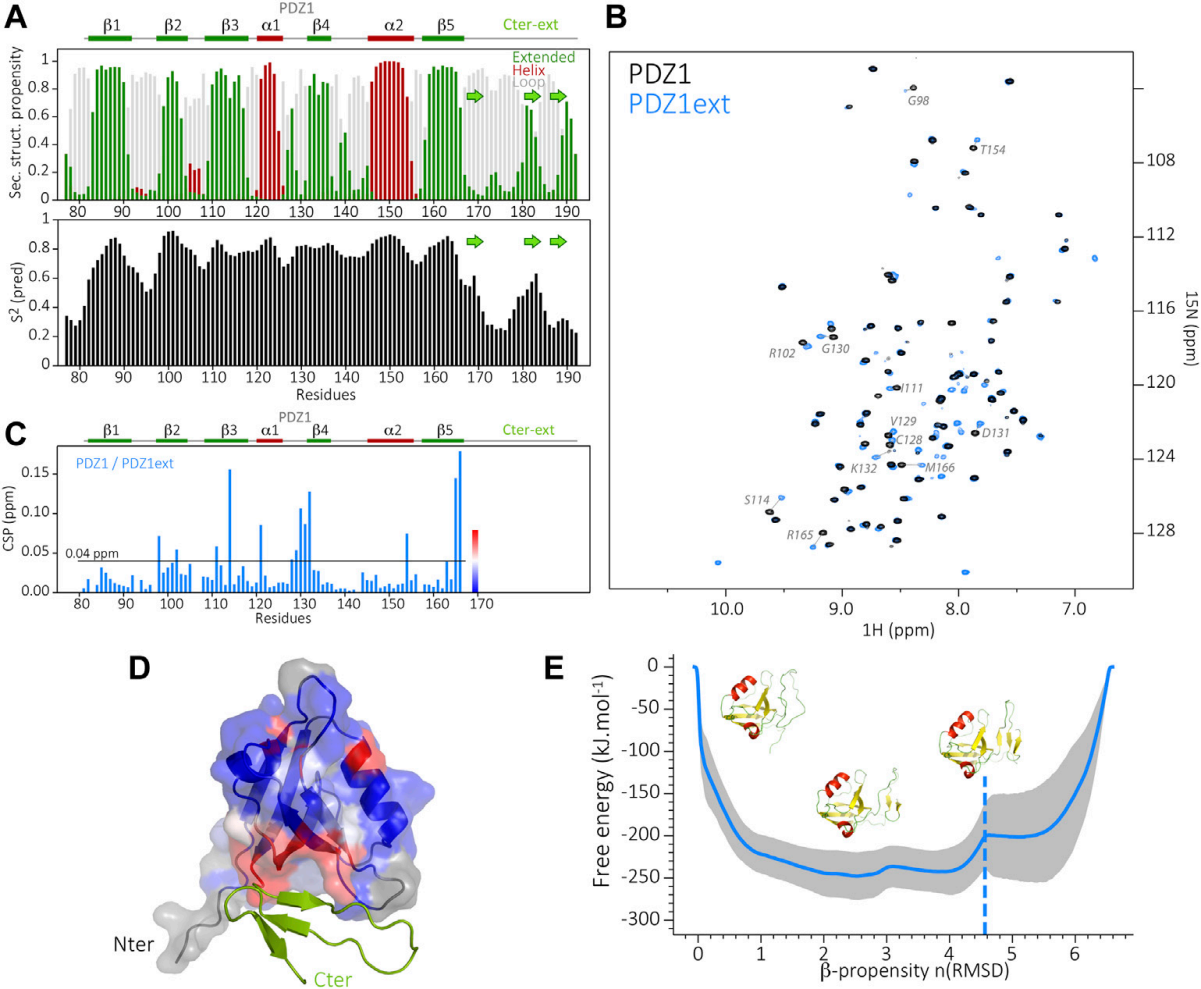
**(A)** Secondary structure prediction from TALOS-N, based on the backbone assignment of PDZ1ext, showing the propensity of β-strands in green and α-helices in dark red (at the top) and the predicted order parameters S^2^ (at the bottom). In the Cter extension, the consensus position of the three b-strands (green arrows) is inferred from the TALOS order parameters and the AlphaFold predicted model. **(B)** Superimposed ^1^H-^15^N HSQC spectra of PDZ1 (black) and PDZ1ext (blue). Assignment is indicated in gray for the most perturbed PDZ1 residues (CSP >0.04 ppm). Blue peaks without assignment correspond to residues of the Cter extension. **(C)** NMR chemical shift perturbations (CSP) on PDZ1 induced by the Cter extension, *i.e.*, between PDZ1 and PDZ1ext. Secondary structure elements are indicated at the top. **(D)** Structure of PDZ1ext AlphaFold model in ribbon, with the PDZ1 domain displayed as surface and the Cter extension in green. CSP induced by the Cter extension are mapped on the AlphaFold model of PDZ1ext. The color coding from blue (no CSP) to red (large CSP) as indicated in **(C)** highlights the packing of the Cter extension onto PDZ1, in agreement with the AlphaFold model. Residues depicted in grey have no associated chemical shift (missing peak in PDZ1ext). **(E)** Energy landscape obtained by metadynamics computing. The blue line indicates the average energy measured for each β propensity across four trajectories. Standard deviation is indicated by the grey outline. Displayed snapshots correspond to conformations extracted from the trajectory. The vertical dashed line indicates the starting point of the simulations.

We produced a ^15^N and ^13^C labeled sample of PDZ1ext to characterize the folding state of the PDZD7 PDZ1 C-terminal extension. We successfully assigned 98%, 99% and 100% of the (H, N), CO and (CA,CB) backbone resonances of PDZ1ext, respectively. Secondary structure analysis derived from HN, N, CA, CB and CO chemical shifts predicts backbone torsion angles consistent with a triple β strand fold of the PDZ1 extension (TALOS-N prediction server; Shen and Bax, 2013) (Figure 2A). These three strands display lower propensities than those in the PDZ core (around 0.6). Consistently, the predicted order parameters are lower than in the rigid secondary structure elements of the core (S^2^_pred_ < 0.6), but higher than expected for a flexible unfolded tail. Moreover, the peak intensities of the β strand extension in the ^1^H-^15^N HSQC spectrum at pH 6.5 are similar to the ones of the PDZ core (data not shown), but are significantly reduced at higher pH (7.4) due to increased HN/H_2_O exchange. Altogether, these results are consistent with the formation of transient C-ter β-strands.

Furthermore, the mapping of Chemical Shift Perturbations (CSP) calculated for backbone resonances between PDZ1 and PDZ1ext constructs highlights an area encompassing β2 to β4 strands and the α1-β4 loop of the PDZ core (Figures 2B,C). This result is in agreement with the model of PDZ1ext predicted by AlphaFold (Jumper et al., 2021) (Figure D), where the triple β strand extension is packed onto the inner leaflet of the PDZ, although a lower confidence level is found in the C-terminal extension (lDDT around 60%, Supplementary Figure S1).

Finally, we used metadynamics (Branduardi et al., 2012) enhanced sampling to depict the free-energy landscape of PDZD7 PDZ1 extension and assess the relative stability of its folded states. We used the Beta Collective Variable (Pietrucci and Laio, 2009) on the β strand extension to enlarge the conformational space sampled for this region. The β strand scoring (bounded between 0 and 1) was computed for each residue of the considered region. The sum of this score thus reflects the overall β propensity of the extension throughout the simulation. The free energy associated with each sampled conformation was calculated (Bonomi et al., 2009). The metadynamics showed that multiple metastable folded states of the β strand extension are explored with a β-propensity between 2 and four for the lowest free-energies, consistent with the partially stable β strands predicted by NMR. In conclusion, the C-terminal extension of PDZD7 PDZ1 adopts a dynamic triple β strand conformation, transiently in contact with the PDZ core.

### Folding of the PDZ2 Extension

**FIGURE 3:**
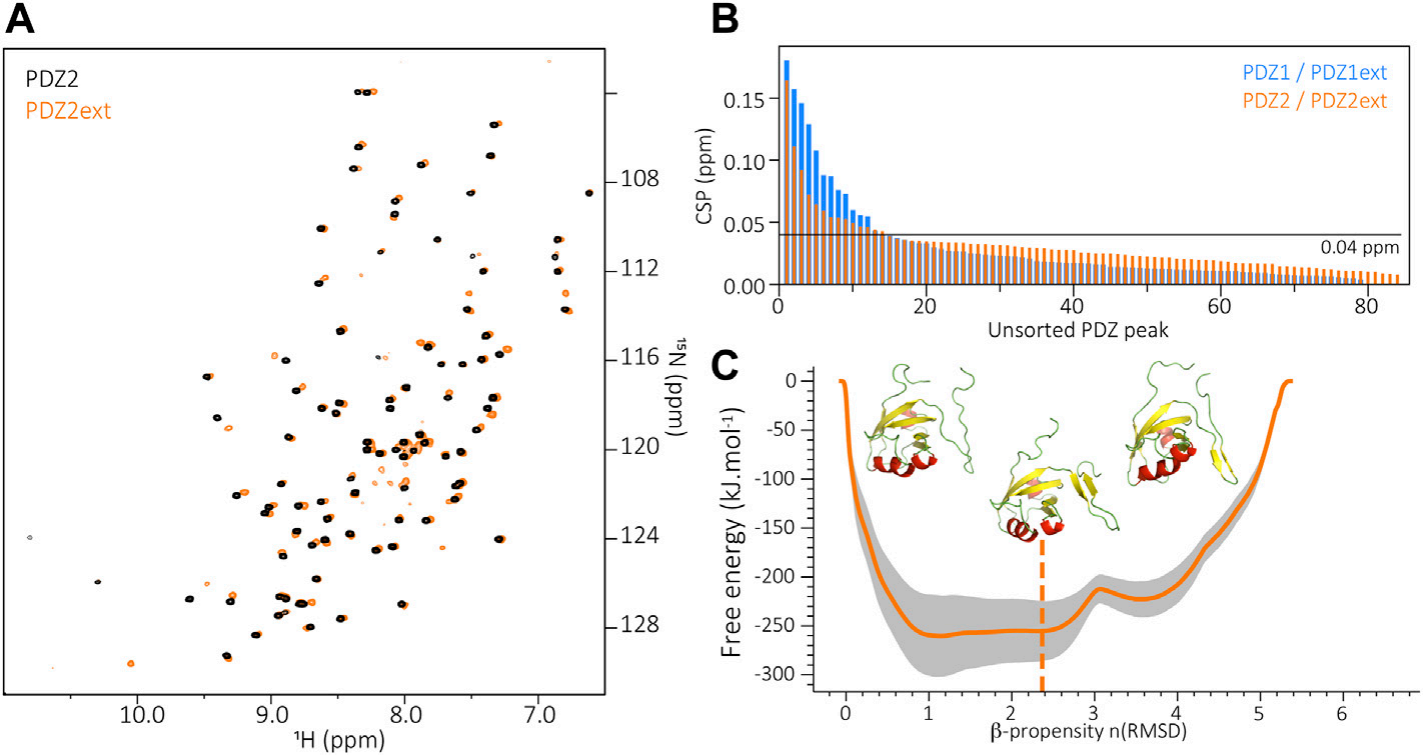
**(A)** Superimposition of PDZ2 (black) and PDZ2ext (orange) ^1^H-^15^N HSQC spectra. **(B)** Unsorted Chemical Shift Perturbations (CSP) measured for each residue of the PDZ core when comparing PDZ1 to PDZ1ext (blue) and PDZ2 to PDZ2ext (orange). **(C)** Energy landscape obtained by metadynamics computing. The orange line indicates the average energy measured for each β propensity across four trajectories. Standard deviation is indicated by the grey outline. Displayed snapshots correspond to conformations extracted from the trajectory. The vertical dashed line indicates the starting point of the simulations.

The biochemical behavior of PDZD7 PDZ2 drastically differs from PDZ1, with or without its C-terminal extension. Rapid aggregation of both purified PDZ2 and PDZ2ext is observed at temperatures higher than 15 °C despite extensive screening of buffer conditions (including buffer, pH, salt and additional ions). The ^15^N-labelled PDZ2 constructs were stable for a few hours only, preventing the acquisition of 3D heteronuclear NMR experiments and the assignment of PDZ2 and PDZ2ext for an analysis at the residue level. Nonetheless, we recorded 2D HSQC spectra on PDZ2 and PDZ2ext samples (Figure 3A) and we observed that the presence of the PDZ2 extension induces perturbations of resonances on the PDZ core comparable in number and amplitude to those detected between PDZ1 and PDZ1ext (Figure 3B). The subsequent CSPs ranked by decreasing order indicate that 16.3% of PDZ2 residues have CSPs higher than 0.04 ppm and 15.3% in the case of PDZ1 in agreement with perturbations of comparable amplitude induced by the extensions on both PDZs. In order to further establish the accessible folding states of the PDZ2 extension, we used the same metadynamics approach as for PDZ1ext and probed its conformational energy landscape (Figure 3C). Again, we observe accessible metastable folded states of the PDZ2 extension, with β propensities between one and four and associated free energies comparable to the ones of PDZ1ext. Altogether, these results suggest an exchange between unfolded and β conformations of PDZD7 PDZ2 extension.

### Supramodular Conformation of the PDZ Tandem

As previously stated, folding of PDZ extensions downstream PDZ1 and PDZ2 domains of harmonin and whirlin is necessary for the supramodular organization of the proteins. In particular, the two N-terminal PDZ domains of whirlin adopt a compact conformation with a hairpin-mediated interface. In PDZD7, our results showed that PDZ1 and PDZ2 also possess C-terminal folded extensions. We collected Small Angle X-ray Scattering (SAXS) data and determined geometrical parameters of the PDZD7 PDZ tandem (P1P2 construct; A81 to G313), with a derived radius of gyration (Rg) of 27.8 Å and interatomic maximum distance (Dmax) of 109.5 Å. These parameters are comparable to what we previously measured on the PDZ tandem supramodule in whirlin (Rg = 27.5Å; Dmax = 115Å) (Delhommel et al., 2017). The estimated molecular weight of 24 kDa derived from the PDZD7 SAXS data is also consistent with the theoretical value of 25.8 kDa. As illustrated by the dimensionless Kratky representation, PDZD7 PDZ tandem adopts a rather compact, yet elongated conformation in solution, similar to the PDZ supramodule of whirlin (Supplementary Figure S2).

**FIGURE 4:**
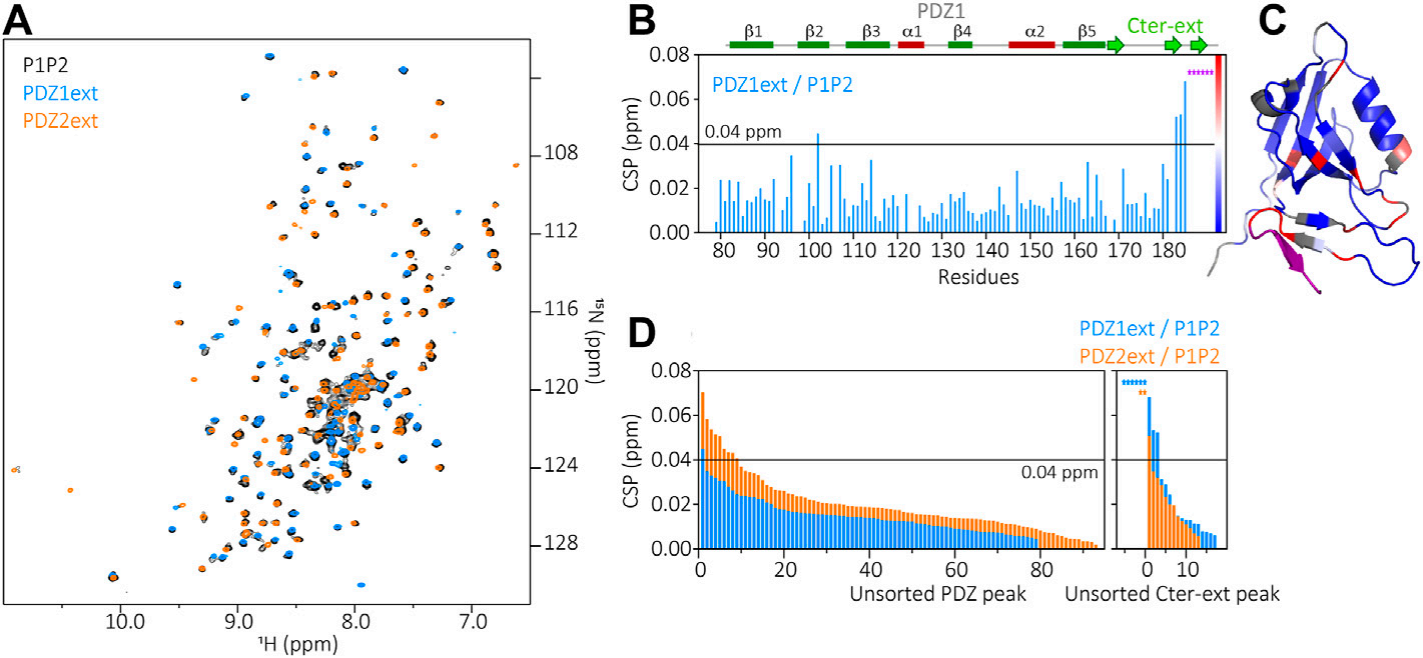
**(A)** Superimposition of P1P2 (black), PDZ1ext (blue) and PDZ2ext (orange) ^1^H-^15^N HSQC spectra. **(B)** Chemical Shift Perturbations (CSP) measured for each residue of PDZ1ext when comparing PDZ1ext to P1P2. Purple stars correspond to residues of the extension which can not be assigned in P1P2 due to large CSP or missing peaks, indicating large changes of environment. Secondary structures are represented above the barplot. **(C)** Mapping of the PDZ1ext/P1P2 CSPs on PDZ1ext AlphaFold model with the color coding indicated in **(B)**. **(D)** Unsorted CSPs measured for each residue when comparing PDZ1ext to P1P2 (blue) and PDZ2ext to P1P2 (orange). Colored stars correspond to residues which can not be assigned in P1P2 due to large CSP or missing peaks, indicating large changes of environment.

The superimposition of HSQC spectra of PDZ1ext, PDZ2ext and P1P2 in Figure 4 show well overlapped peaks of the isolated PDZ domains and of the PDZ tandem (Figure 4A). This results in small CSPs between P1P2 and each PDZ domain, indicating only slight changes of environment of each PDZ domain in the context of the tandem. Only the last 2 β strands of PDZ1 extension are significantly perturbed in the PDZ tandem (CSPs >0.04 ppm) (Figures 4B,C), with reduced peak intensities likely reflecting a loss of flexibility and a potential stabilization of the C-terminal extension. Similarly, only few residues of the second PDZ exhibit significant CSPs (>0.04 ppm) in the presence of the PDZ1 (Figure 4D). In conclusion, the two N-terminal PDZ domains of PDZD7 are not independent in solution, with an interplay likely mediated by folded C-terminal sequences and with little perturbations relayed to the core of each PDZ domain. Altogether, the PDZ tandem of PDZD7 appears to have a similar behavior as the one of whirlin, which is in equilibrium between partially opened and closed conformations.

### Affinity of the PDZ Domains and Tandem Towards ADGRV1pbm

We monitored the interaction between PDZD7 constructs and a peptide (ADGRV1pbm) corresponding to the last 13 residues of ADGRV1, encompassing its PBM (sequence VELRRIPIADTHL_-COOH_; PBM underlined). We measured apparent Kd values of our constructs towards ADGRV1pbm using several biophysical methods (NMR, fluorescence, MicroScale Thermophoresis (MST)) (Table 1; Supplementary Figure S3–S5). First, we showed that the isolated PDZ1 and PDZ2 of PDZD7 bind to ADGRV1pbm with weak affinities of 1313 and 313 µM, respectively. However, addition of their respective extensions induces a dramatic increase of the affinity towards ADGRV1pbm, with Kd values of 47 µM for PDZ1ext and 3.1 µM for PDZ2ext. In both cases, these extensions appear critical for the interaction, with PDZ2ext being the preferential binder. As monitored by NMR, addition of ADGRV1pbm to PDZ1ext induces large chemical shift changes for residues in the canonical binding site defined by the β2 strand and α1 helix and the loss of peaks corresponding to residues in the PDZ extension due to intermediate exchange processes. This underlines a change in dynamics of the pre-formed triple strands upon peptide binding. Further shifts of residues from the β3 and β4 strands also suggest a potential stabilization of the interface between the folded extension and the inner leaflet of the PDZ core. The contribution of this extension to the increase of affinity towards ADGRV1pbm could thus arise from direct contacts with the peptide as well as distal structural/dynamic perturbations of the PDZ core. Altogether, these results support a structural contribution of the hairpin sequence of PDZD7 PDZ1 to the interaction with ADGRV1pbm.

**TABLE 1:** Kd values measured for PDZD7 PDZ constructs in interaction with the last 13 residues of ADGRV1. PDZ1 and PDZ1ext by NMR titration; PDZ2 by MST titration; PDZ2ext and P1P2 by fluorescence titration). Errors indicated with * derive from the quality of the fit. For PDZ1 and PDZ1ext, errors derive from the standard deviation of measured Kd values for at least ten significantly shifting peaks.

Construct	Kd
PDZ1	1,313 ± 81 µM
PDZ2	313 ± 52 µM*
PDZ1ext	47 ± 6 µM
PDZ2ext	3.1 ± 0.2 µM*
P1P2	1.6 ± 0.3 µM*

We also showed that PDZ2ext has a similar affinity (Kd = 3.1 µM) for ADGRV1pbm compared to the PDZ tandem (P1P2; Kd = 1.6 µM), but 15-fold higher than PDZ1ext (Kd = 47 µM). Taking advantage of the good superimposition of the isolated PDZ spectra on the tandem spectrum, we followed the saturation of each PDZ in the tandem as a function of ADGRV1pbm concentrations (data not shown). We confirmed that both PDZ domains in the tandem bind to ADGRV1pbm and that PDZ2 is saturated at lower ligand concentration than PDZ1, in agreement with the affinities measured for the isolated domains. Finally, we assessed if the presence of ligand further stabilizes a compact conformation of the P1P2 supramodule using SAXS and Analytical UltraCentrifugation (AUC) (Supplementary Figure S6). For the bound P1P2, we derived a Rg of 27.4 Å and a Dmax of 104 Å from SAXS curves, as well as a sedimentation coefficient of 1.7s from AUC. Compared to the unbound PDZ tandem (Rg = 27.8 Å, Dmax = 109.5 Å, S = 1.6s), these results reflect a minor, if significant, compaction of the supramodule without major structural reorganization of the protein.

### Stability of the PDZ Domains and Tandem

We measured the thermostability of PDZD7 constructs using nanoDifferential Scanning Fluorimetry (nanoDSF). As previously observed during the production and biochemical/biophysical characterizations of the domains, PDZ1ext is drastically more stable than PDZ2ext, with melting temperatures of 61.6°C and 38.5°C, respectively. Interestingly, the binding of ADGRV1pbm strongly increases the thermal stability of the unstable PDZ2ext (Tm increased from 38.5°C to 47.2°C), while the binding has no significant effect on the more stable PDZ1ext (Tm increased from 61.6°C to 61.8 °C) (Table 2). The melting temperature of P1P2 also seems to be driven by the PDZ2, with a low Tm for the unbound form (41°C) and a strong stabilization upon ADGRV1pbm binding (Tm of 57.2°C). Overall, these results showed that ADGRV1pbm, primarily interacting with the second PDZ domain, largely stabilizes the PDZ supramodule.

**TABLE 2:** Melting temperatures (TM) measured for PDZD7 PDZ constructs, without ligand (unbound) or in complex with the last 13 residues of ADGRV1 (bound).

Construct	Tm (Unbound)	Tm (Bound)
PDZ1ext WT	61.6°C	61.8°C
PDZ1ext G103R	56.8°C	56.3°C
PDZ2ext WT	38.5°C	47.2°C
PDZ2ext G228R	∼30°C	∼30°C
P1P2 WT	41°C	57.2°C
P1P2 G103R	34.9°C	53.7°C

### Other Determinants of the PDZD7/ADGRV1 Interaction

The P1P2 tandem of PDZD7 is preceded by 80 residues with no predicted secondary or tertiary structure, but which could contribute to the interaction with ADGRV1. Similarly, the 152 residues of ADGRV1 cytoplasmic domain upstream its PBM exhibit several stretches of conserved positions and could participate in the interaction. We first aimed at identifying potential secondary structures in both PDZD7 N-terminal region and ADGRV1 cytoplasmic domain. To this end, we produced both an extended PDZ1ext construct, including the N-terminus of the protein (N-PDZ1ext construct; M1-E192) and the whole ADGRV1 cytoplasmic domain of 152 residues.

On the overlay of PDZ1ext and N-PDZ1ext HSQC spectra, there is no significant chemical shift changes on PDZ1ext due to the presence of the N-terminal region (CSPs <0.04 ppm). The latter is characteristic of a disordered region, with about 70 peaks (80 expected) of poor dispersion of HN chemical shifts (8–8.5 ppm) (Supplementary Figure S7). This supports that the N-terminal 80 residues are unfolded and independent of the downstream PDZ1ext domain. We confirmed using SAXS experiments that the upstream N-terminal region is extended, flexible and behaves independently from the folded PDZ1 domain (Supplementary Figure S2). For ADGRV1 cytoplasmic domain, the HSQC spectrum also displays a weak dispersion of signals (8–8.5 ppm), corresponding to a predominantly disordered protein (Supplementary Figure S8) and we confirmed the absence of stable secondary structures in solution using circular dichroism that showed a spectrum largely governed by random coil conformations (data not shown). We then aimed at deciphering the potential contribution of these flexible sequences to the interaction between ADGRV1 and PDZD7 (Table 3). We used the entire cytoplasmic domain of ADGRV1 as partner of PDZD7 in NMR and fluorescence titrations experiments. First, we quantified the interaction with PDZ1ext, obtaining an apparent Kd value of 41 ± 14 µM comparable to the value 47 ± 6 µM obtained with the ADGRV1pbm peptide. Similarly, we used the N-terminal PDZD7 sequence N-PDZ1ext and measured Kd values of 241 ± 115µM and 270 ± 21 µM for ADGRV1 cytoplasmic domain and PBM peptide, respectively. In addition, no significant CSPs have been observed in the N-terminal sequence upstream PDZ1 upon ADGRV1 binding. The presence of the long unfolded N-terminus region induces a 6-fold decrease in affinity for ADGRV1 (PBM peptide or cytoplasmic domain) that might be due to an entropic cost on the PBM binding. Finally, we measured Kd values in the few micromolar range between the tandem P1P2 and the whole cytoplasmic domain and the last 13 residues of ADGRV1 by fluorescence experiments. Altogether, these results showed that the interaction between ADGRV1 cytoplasmic domain and PDZD7 do not involve other determinants besides the PBM-PDZ interaction.

**TABLE 3:** Kd values measured for PDZD7 constructs against ADGRV1 whole cytoplasmic domain (cyto) or last 13 residues (peptide) (PDZ1ext and N-PDZ1ext by NMR titration; P1P2 by fluorescence titration). For fluorescence, the error derives from the quality of the fit (marked as *). For NMR, errors derive from the standard deviations of the measured Kd values for at least ten significantly shifting peaks.

Construct	Kd (Cyto)	Kd (Peptide)
PDZ1ext	41 ± 14 µM	47 ± 6 µM
N-PDZ1ext	241 ± 115 µM	270 ± 21 µM
P1P2	8.1 ± 1.1 µM*	1.6 ± 0.3 µM*

### Pathogenicity of the Mutants

Two deafness-causing mutations of the same amino acid substitution are located at the same position in the binding groove of the first (G103R) and second (G228R) PDZ domains in PDZD7 (Booth et al., 2015). We first assessed by nanoDSF the effect of these mutations on the thermal stability of PDZD7 constructs (Table 2).

The mutation in PDZ1 (G103R) induces a significant destabilization of both isolated PDZ domains (PDZ1ext, Tm decreased by 4.8°C for the apo form) and PDZ tandem (P1P2, Tm decreased by 6.1°C for the apo form). The second mutation (G228R) markedly destabilizes the isolated PDZ2ext, with a slow transition of fluorescence ratio (330/350 nm) initiated below 15°C, indicating progressive unfolding of the protein at room temperature, with a Tm that can be roughly estimated in the 30°C range (Supplementary Figure S9). As a consequence, we could not express the PDZ tandem carrying the G228R mutation. Globally, both deafness mutations significantly decrease the thermostability of PDZ domains with a more pronounced effect on PDZ2, initially more unstable. Interestingly, addition of ADGRV1pbm peptide still stabilizes the P1P2 tandem when the PDZ1 is mutated (by 19°C in Tm), consistent with a functional binding site in the PDZ2 and an overall melting temperature of P1P2 driven by this second PDZ.

We characterized the potential effect of the two substitutions on the interaction with ADGRV1pbm using a panel of techniques. First, we confirmed by NMR that PDZ1ext G103R is well-folded, with significant perturbations (CSPs >0.04 ppm) only detected for residues spatially close to the G103R mutation (Supplementary Figure S10). In addition, we observed an increase in peak intensity in the C-terminal β strands facing the mutated residue, suggesting a potential higher flexibility of the extension. This G103R substitution results in a 25-fold decrease in affinity (Kd from 47 ± 6µM to 1,190 ± 140 µM for WT and G103R PDZ1ext, respectively) showing that this mutation interferes with the binding of PDZD7 to its partners through PDZ1ext. A similar affinity in the mM range has been determined for PDZ1 lacking the β strand extension (Kd of 1,310 ± 80 µM), suggesting that the G103R mutation most likely affect the interface between the PBM pocket and the C-terminal extension, making it incompetent for binding. The substitution of a small, neutral side chain (glycine) by a large, cationic side chain (arginine) residue could also induce steric hindrance in the binding pocket. Similar local perturbations are observed in the P1P2 G103R tandem, but WT and G103R mutated constructs have similar affinities measured by fluorescence, with Kd values of 1.6 ± 0.3 µM and 1.9 ± 0.4 µM respectively. This first mutation thus alters direct PBM binding to the first PDZ, but has no long-distance impact on the binding of ADGRV1pbm to the second PDZ. This result confirms the main role of PDZ2 in the interaction with ADGRV1. We used SAXS to monitor global conformational changes of the PDZ supramodule carrying the G103R mutation. We calculated Rg = 28 Å and Dmax = 118.2 Å for the mutant tandem without ligand, and Rg = 27.7 Å and Dmax = 123.6 Å in presence of ADGRV1pbm peptide. These values are slightly larger than in the WT P1P2, which might indicate a shift of the closed/open conformer equilibrium toward a relaxed state of the tandem. This could result from the increased flexibility of the PDZ1 β strand extension required to form the interface in the supramodule.

We could not measure the interaction between PDZ2 G228R and ADGRV1pbm, due to a low yield and severe aggregation of the mutated domain during biophysical experiments, consistent with its low melting temperature. For the same reasons, we are unable to produce the PDZ tandem carrying the G228R substitution, preventing us from measuring its affinity for ADGRV1pbm.

To conclude, the substitution G103R prevents the first domain of the tandem from binding ADGRV1, but does not affect the binding capacity of the second domain. On the other hand, it significantly alters the overall thermostability of the supermodule even in complex with ADGRV1pbm. The other substitution G228R in PDZ2 has an even greater effect by targeting the more unstable PDZ domain and the best ADGRV1 binder in the tandem.

### Full-Length Proteins in Cell Assays

**FIGURE 5:**
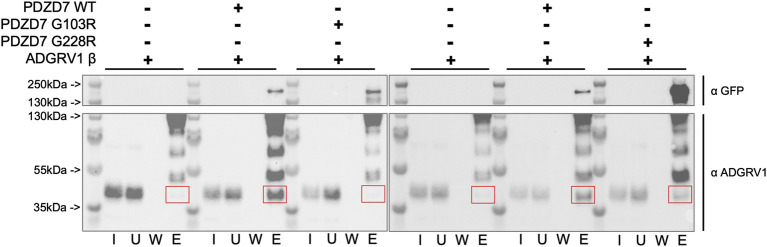
Western blot detection of ADGRV1 β subunit (prey) pulled down by PDZD7-GFP (bait) constructs from HeLa cell lysates. PDZD7 constructs are detected with an antibody directed against the fused GFP. ADGRV1 is detected using an antibody directed against its cytoplasmic domain. For each condition, fractions corresponding to the input (I), unbound fraction (U), wash (W) and elution (E) are loaded. Red squares indicate the expected position for ADGRV1 β in the elution fraction.

Finally, we used pull-down assays to test the interaction between full-length PDZD7 wild-type and mutants (in fusion with a C-terminal GFP), and ADGRV1 β subunit, encompassing the seven transmembrane segments and cytoplasmic domain. Proteins are expressed separately in HeLa cells and lysates are mixed after sonication. Addition of G sepharose beads coupled to an anti-GFP antibody (see Material and methods) allowed to bind PDZD7. Three experiments were performed in parallel for each mutant; first a pull-down of ADGRV1 without bait to rule out unspecific binding (negative control), then with WT PDZD7 (positive control) and finally with the considered mutant. For each experiment, the same samples corresponding to the input, unbound, wash and elution fractions are loaded in equal amounts into two twin gels (top and bottom parts). Each gel is used to detect the presence of either PDZD7-GFP (Figure 5, top part) or ADGRV1 (Figure 5, bottom part) on the beads after washing by western blotting. Each mutant is therefore tested in independent experiments in comparison with the wild-type (Figure 5 left part for G103R and right part for G228R). In both cases, little to no ADGRV1 is detected bound to the beads without bait, while a clear band is observed with WT PDZD7 as bait. Thus, full-length PDZD7 interacts with ADGRV1 β subunit, as expected. When using either G103R or G228R mutants of PDZD7, a decrease of ADGRV1 quantity bound to the beads was observed in comparison with wild-type PDZD7, similar to the experiment performed without bait, although the amount of bait on the bead was comparable (Supplementary Figure S11). Therefore, both G103R or G228R variants impair binding of full-length PDZD7 to ADGRV1. To note, the two PDZD7 variants are expressed in lower quantities compared to the wild-type when transfecting an equal amount of cells, and have a higher tendency for degradation, resulting in multiple bands of low molecular weight in the western blot (Supplementary Figure S11). This observation reflects the lower stability of the variants paired with the loss of binding capacity to ADGRV1, in agreement with the results obtained *in vitro* using purified mutated PDZ constructs.

## Discussion

PDZD7 and ADGRV1 are the main candidate proteins for regulating the signaling associated with the Usher2 complex. PDZD7 is primarily a scaffolding protein, with several protein-protein interaction domains (three PDZ domains and one HHD) likely recruiting several partners at the ankle links anchoring sites (Du et al., 2018). Interestingly, PDZD7, but not its homologue whirlin, downregulates ADGRV1 GPCR activity in cellular models in a PDZ-PBM dependent manner (Hu et al., 2014).

Here we show that both N-terminal PDZ1 and PDZ2 domains of PDZD7 can interact with the C-terminal PDZ binding motif of ADGRV1. Our results highlight a critical contribution of atypical PDZ domain C-terminal extensions to the binding, tuning affinity towards partners. Indeed, the presence of these extensions results in a ∼20 to 100 fold increase in affinity for each PDZ towards ADGRV1pbm. In addition, the ADGRV1 motif preferentially interacts with PDZD7 PDZ2, with a ∼15 fold higher affinity (Kd = 3.1 µM) compared to PDZ1 (Kd = 47 µM). Therefore, ADGRV1pbm binds to PDZ2 in the higher range of affinity than what is commonly found for PDZ domain interactions (1-100 µM). As monitored by NMR on PDZ1, the C-terminal extension of the domain adopts an original three β strands conformation in solution, that is at least partially in contact with the core of the PDZ (β2, β3 strands), consistent with AlphaFold’s prediction. Interestingly, addition of the ADGRV1pbm peptide induces a change in dynamics of the extension, potentially favoring the folded state, and a compaction onto the PDZ core. The increase of affinity induced by the extension could arise from a modulation of the dynamics of the PDZ core in favor of the bound state, or from additional contacts between the extension and the partner upstream of its PBM, as observed in the homologous protein harmonin in complex with the protein SANS (Yan et al., 2010) (PDB entry 3k1r). PDZD7 PDZ2 also possesses a functional C-terminal extension responsible for a drastic increase in affinity of the PDZ towards ADGRV1pbm. We showed that the C-terminal extension of PDZ2 has an energy minimum corresponding to a folded β hairpin conformation. Furthermore, the presence of this extension induces perturbations of the ^1^H-^15^N spectra of the PDZ2 similar to the ones observed in PDZ1, suggesting comparable contacts with the PDZ core. Lastly, complex formation with ADGRV1pbm also leads to a change in dynamics of the extension for both PDZ, resulting in a loss of signals by NMR. Altogether, these results support an accessible 2-3 β strands conformation of each PDZ extension in solution.

In the homologous proteins whirlin and harmonin, PDZ extensions mediate the formation of supramodules. In harmonin, only the first PDZ possesses a C-terminal hairpin extension, mediating an interface with the preceding HHD domain (Yan et al., 2010). In whirlin, the N-terminal PDZ tandem is in equilibrium between open states and a close conformation. In this compact supramodule, each PDZ extension folds as a β hairpin packed onto the inner leaflet of its adjacent PDZ. The two hairpins then form the interface between the two PDZs (Delhommel et al., 2017) without direct contact between the cores of the two domains. PDZD7 PDZ tandem also adopts a rather compact and elongated conformation in solution, possibly in exchange with more open conformations, with SAXS parameters comparable to the ones of whirlin. Addition of ADGRV1pbm peptide seems to favor the closed conformation of the PDZ tandem through the stabilization of the β extensions. However, the tandem conformation has limited effect on affinity towards the partner when compared to PDZ2 alone (Kds of 1.6 ± 0.3 µM and 3.1 ± 0.2 µM, respectively). PDZD7 PDZ tandem still exhibits two functional binding sites, allowing it to potentially bind two ADGRV1pbm in the highly confined environments of the stereocilia, which would be compatible with the high protein densities found at the ankle links anchoring sites. PDZD7 PDZ1 could also have other natural binders instead of ADGRV1. PDZD7 PDZ2 displays a histidine in position 1 of the α2 helix lining the binding groove, canonically interacting with type 1 (-S/T-X-Φ_-COOH_,X being any residue and Φ a hydrophobic residue) PBM such as the one of ADGRV1 (-**T**HL_-COOH_). By contrast, PDZD7 PDZ1 unusually exhibits a methionine at position 1 of its α2 helix (only four amongst the 266 human PDZ domains), more suitable for type 2 (-Φ-X-Φ_-COOH_) PBM binding. Therefore, PDZD7 PDZ1 might recruit other partners in the vicinity of ADGRV1 (Du et al., 2018) or more generally promote the assembly of ternary proteins within the Usher2 complex. Furthermore, the difference in thermostability of the two PDZ domains could also underlie distinct functions in protein assemblies. PDZ1 is highly stable with and without partner. Conversely, the salvageability of PDZ2 instability by the binding of ADGRV1 PBM possibly regulates temporal maintenance of the protein by promoting its recycling in the absence of ligand. The temporal expression of ADGRV1 and PDZD7 at the ankle links has been poorly described and events triggering expression, persistence and degradation of the two proteins remain unclear. PDZD7 destabilization in absence of PDZ2 ligand might help coordinate disassembly of the complex in maturing hair cells.

To probe for additional determinants of interaction between PDZD7 and ADGRV1, we enlarged our study to PDZD7 N-terminal region (80 first residues upstream the PDZ tandem) and ADGRV1 whole cytoplasmic domain (152 intracellular residues). We confirmed that PDZD7 residues upstream of the tandem do not exhibit tertiary structures, are largely extended and flexible in solution and do not induce perturbations onto the PDZ1 core. Similarly, we have shown that the isolated cytoplasmic domain of ADGRV1 is mostly unfolded and flexible. In terms of interaction, we measured similar affinities for ADGRV1 PBM or whole cytoplasmic domain with the PDZD7 constructs we tested. In conclusion, the PBM of ADGRV1 and the PDZ tandem of PDZD7 are the only determinants of interaction between the two proteins in our experiments. Altogether, our results highlight the critical role of flanking regions in PDZ interactions and supramodular organization, which has not been systematically documented thus far. Interestingly, five out of the six PDZ located in the N-terminal regions of the three paralogous proteins harmonin, whirlin and PDZD7 have their binding capacity modulated by domain extensions. These sequences have been conserved during evolution and could play a unique role as modulator of affinity and specificity. More generally, PDZ domains are known to form hetero and homotypic supramodules. One of the most extensively studied supramodule is the three-domains PDZ3-SH3-GK from the protein PSD-95 (Laursen et al., 2020). In addition, about 40% of human PDZ domains have predicted secondary structures within 50 residues of their C-termini (Wang et al., 2010). However, available experimental data do not reflect such propensity. Here our results highlight the need to include these predicted extensions in structural studies, as well as in high-throughput screening assays knowing their potential role on PDZ stabilization and binding properties. A thorough bioinformatics study of the PDZ domain family, aimed at identifying conserved and co-evoluted stretches of residues outside of their canonical fold, could already unveil several regulatory mechanisms for protein-protein interactions, scaffolding and signaling mediated by PDZ containing proteins.

The regulation of GPCR trafficking by PDZ-containing proteins has been well documented (Dunn and Ferguson, 2015). However, to our knowledge, a regulation of GPCR activity through a PDZ/PBM interaction is unique with the ADGRV1/PDZD7 molecular assembly, but the underlying molecular mechanisms remain elusive. ADGRV1 cytoplasmic domain might behave differently in the context of the entire GPCR β subunit, including intracellular loops and plasma membrane, opening other regulatory mechanisms. The regulation by PDZD7 could also arise from direct interaction with proteins mediating GPCR signaling (G protein, arrestin), or by recruiting other G protein signaling inhibitors such as members of the Regulators of G-protein Signaling (RGS) family (Masuho et al., 2020). Such regulation of ADGRV1 by PDZD7 could be affected by pathogenic mutations identified in patients. We have characterized the effect of two autosomal recessive non-syndromic hearing loss variants of PDZD7, with glycine to arginine substitutions in the lining groove of both the first (G103R) and second (G228R) PDZ domains. By probing the thermostability, folding, and binding capacity of the mutated PDZ domains, we showed that each mutation induced a decrease in thermostability of the protein, as well as a decrease of affinity towards ADGRV1. Consistently, our pull-down experiments showed a loss of binding capacity of the full-length PDZD7 to ADGRV1 β subunit with the two variants, likely abrogating regulatory potential. We propose that the destabilization of PDZD7 by the two variants could affect both the formation of Usher2 complex and the regulation of ADGRV1, and induce physiopathogenesis. The effect of these variants on Usher 2 proteins localization in hair cell stereocilia, and ADGRV1 mediated signaling remains to be characterized.

## Data Availability

The original contributions presented in the study are included in the article/Supplementary Material, further inquiries can be directed to the corresponding author.
