# Supplementary material for: Deciphering the Molecular Interaction Between the Adhesion G Protein-Coupled Receptor ADGRV1 and its PDZ-Containing Regulator PDZD7
Source: Front Mol Biosci. 2022 Jun 28;9:923740. doi: 10.3389/fmolb.2022.923740 (PMC9274004; doi:10.3389/fmolb.2022.923740)
Supplement: Supplementary file 1 [file DataSheet1.pdf]

Deciphering the molecular interaction between the adhesion G protein-coupled receptor ADGRV1 and its PDZ-containing regulator PDZD7

Baptiste Colcombet-Cazenave[1,4], Florence Cordier[2], Yanlei Zhu[1], Guillaume Bouvier[2], Eleni Litsardaki[1], Louise Laserre[1], Marie S. Prevost[1], Bertrand Raynal[3], Célia Caillet-Saguy[1], Nicolas Wolff[1]*

[1]Channel Receptors Unit, UMR CNRS 3571, Institut Pasteur, Université de Paris, 75015 Paris, France.
[2]Structural Bioinformatics Unit, CNRS, UMR 3528, Institut Pasteur, Université de Paris, Paris, France
[3]Molecular Biophysics Platform-C2RT, CNRS, UMR 3528, Institut Pasteur, Université de Paris, Paris, France
[4]Complexité du Vivant, Sorbonne Université, 75005 Paris, France

*** Correspondence:**

Corresponding authors. E-mail: nicolas.wolff@pasteur.fr

Supplementary Material

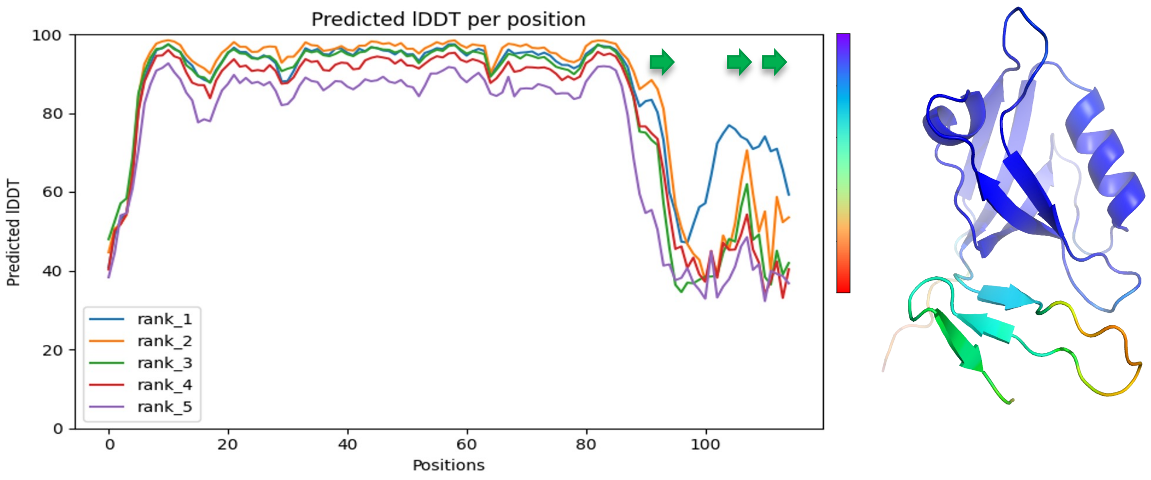

Supplementary 1: Alphafold model obtained for the PDZ1ext construct of PDZD7 (rank 1 displayed). Predicted IDDTs are mapped on the model, highlighting a lower confidence for the triple β strand extension than the PDZ core.

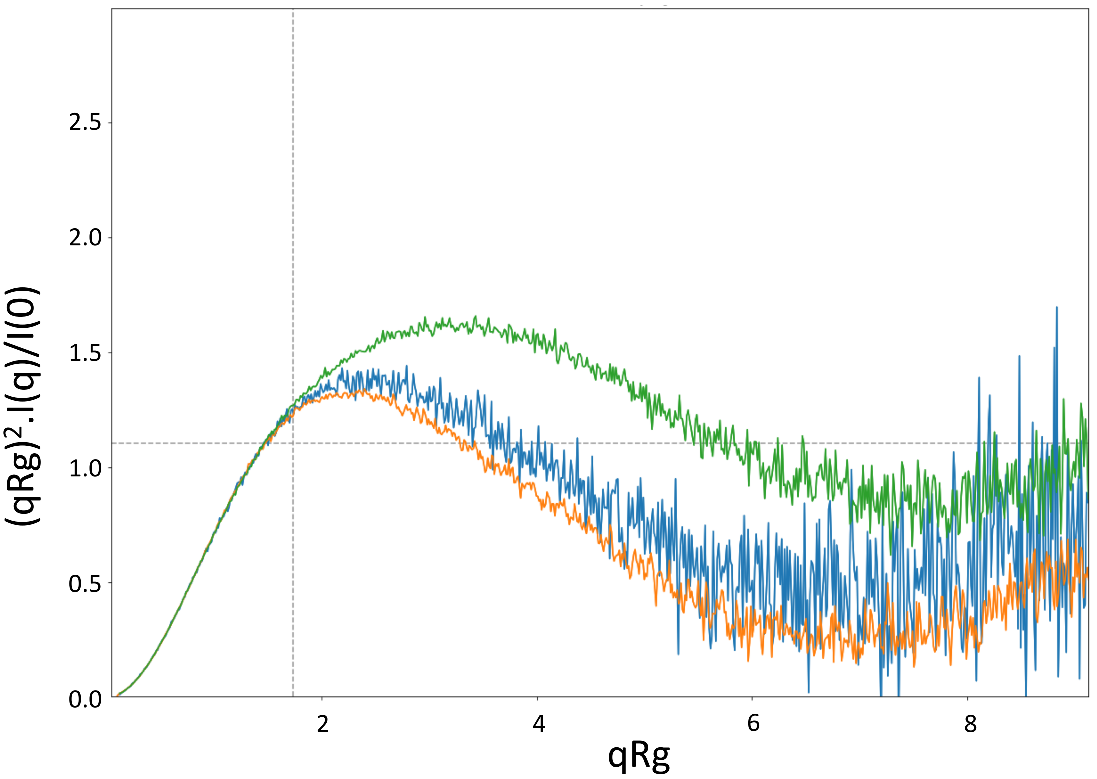

Supplementary 2: Dimensionless Kratky plots of the P1P2 tandem of whirlin (colored in orange), of the P1P2 tandem of PDZD7 (colored in blue) and of the N-PDZ1ext of PDZD7 (colored in green). The dashed lines indicate the peak position (~ 1.7) and peak height (~ 1.1) for a perfectly globular protein.

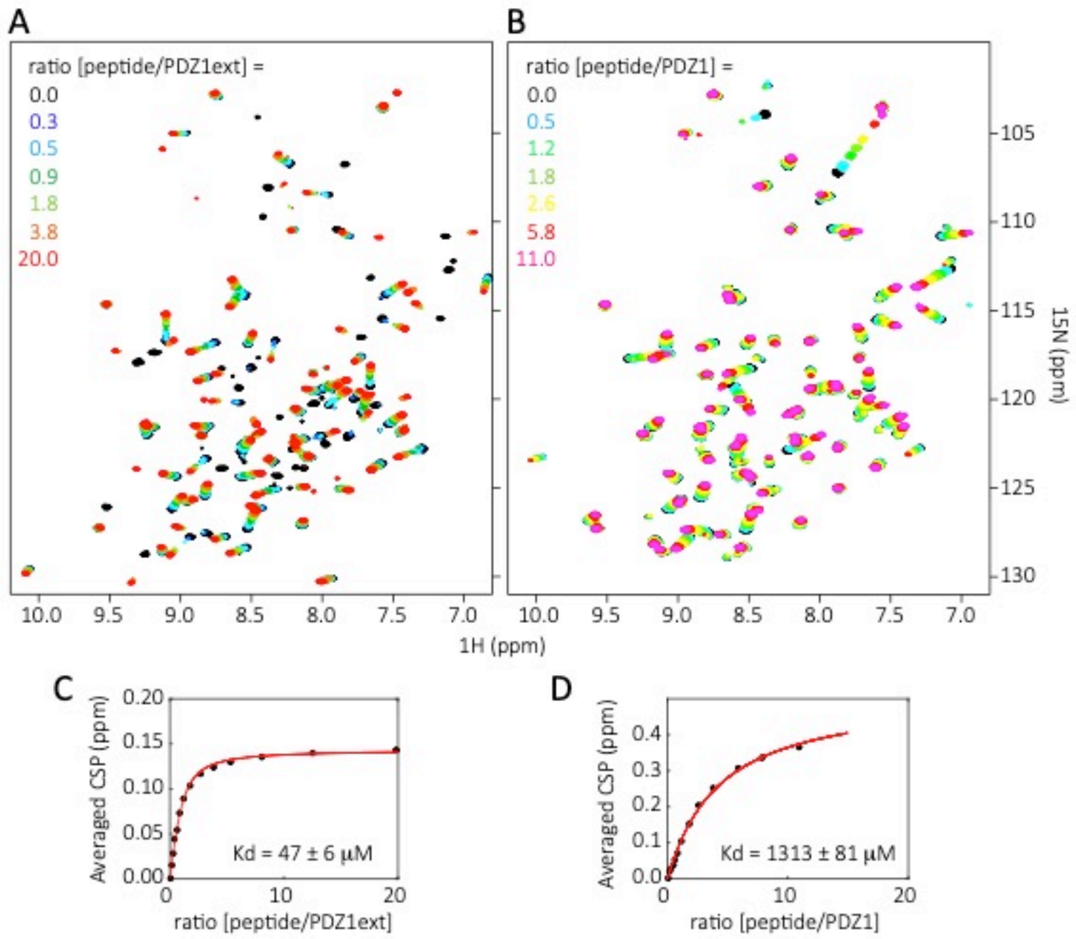

Supplementary 3: Determination of the dissociation constants (Kd) of ADGRV1pbm/PDZ1ext (C) and ADGRV1pbm/PDZ1 (D) interactions: the averaged CSP (over about 10 significantly shifting peaks) are plotted as a function of the molar ratio (peptide/PDZ) and fitted using nonlinear regression and assuming a simple complex formation model. The error on Kd values comes from the standard deviation of Kd calculated from individual fits on each of the significantly shifting peaks. Raw data are indicated by dots and fits by red curves.

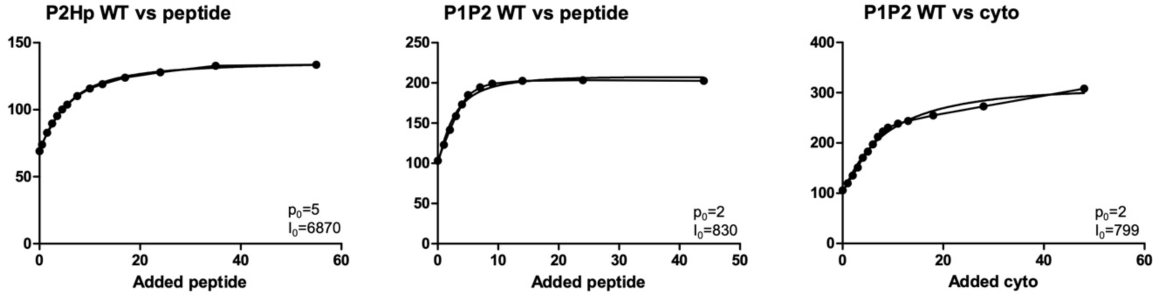

Supplementary 4: Fluorescence titration of P2HpWT, P1P2 WT with ADGRV1pbm peptide, and P1P2WT with ADGRV1 cytoplasmic domain. Raw data are indicated by connected points and fits by curves.

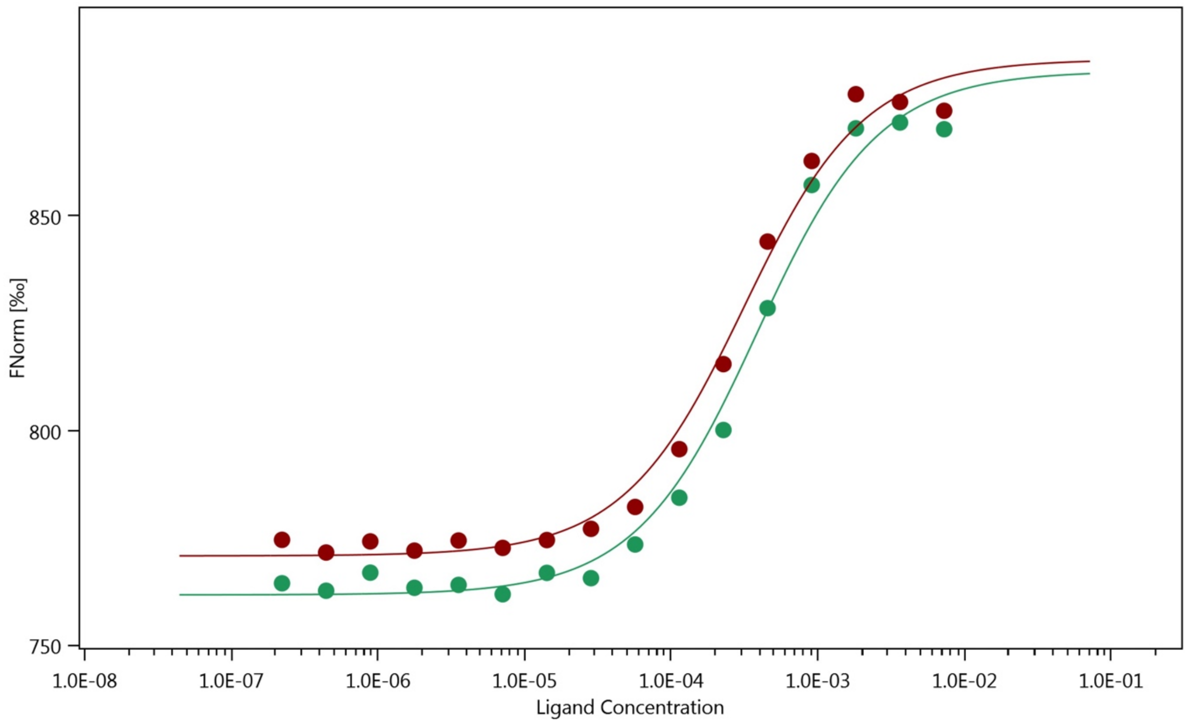

Supplementary 5: MicroScale Thermophoresis titrations (duplicate) of P2 WT with ADGRV1pbm peptide. Raw data are represented by dots and fits by curves.

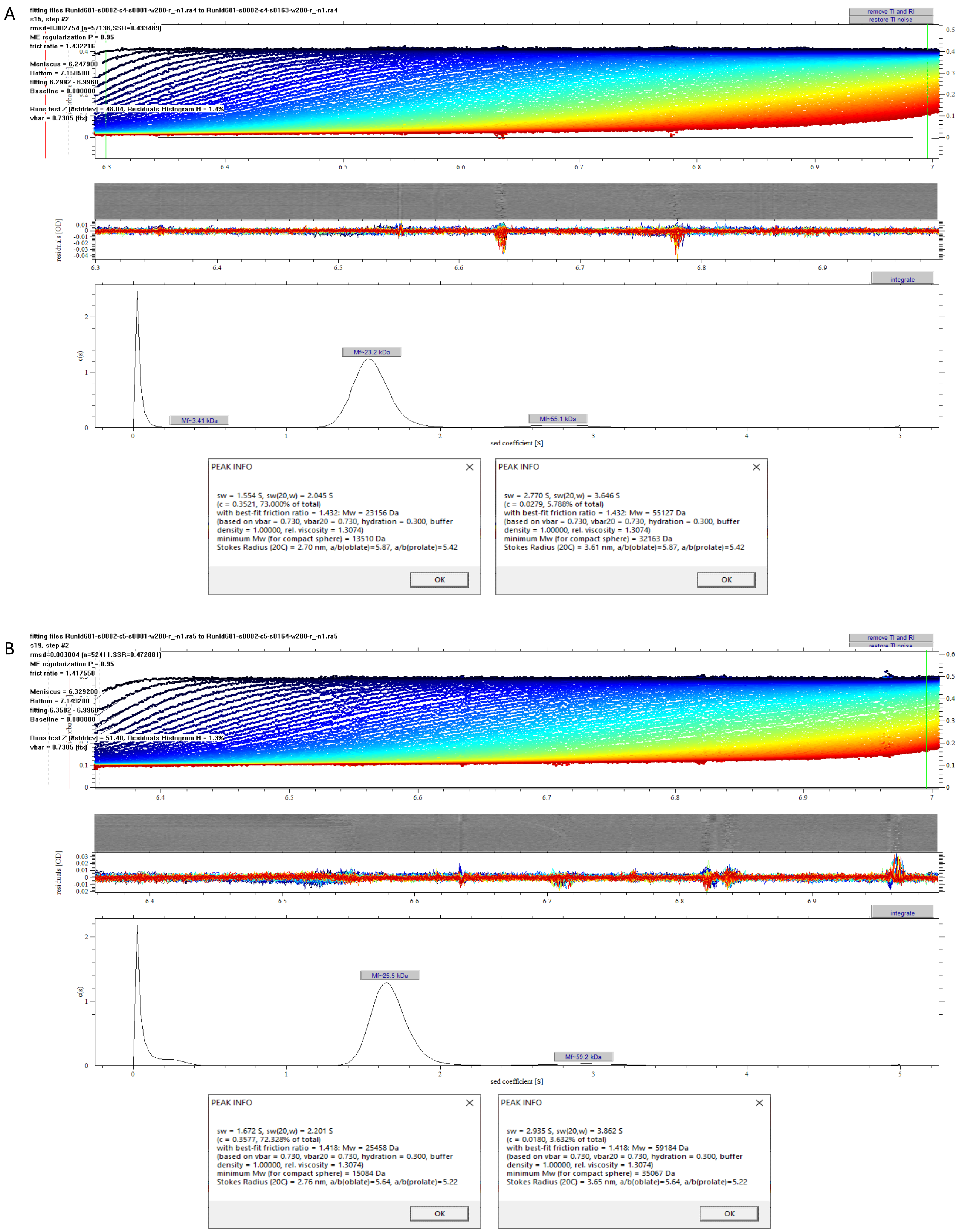

Supplementary 6: Analytical UltraCentrifugation data obtained for PDZD7 P1P2 without ligand **(A)** and in complex with the last 13 residues of ADGRV1 **(B)**.

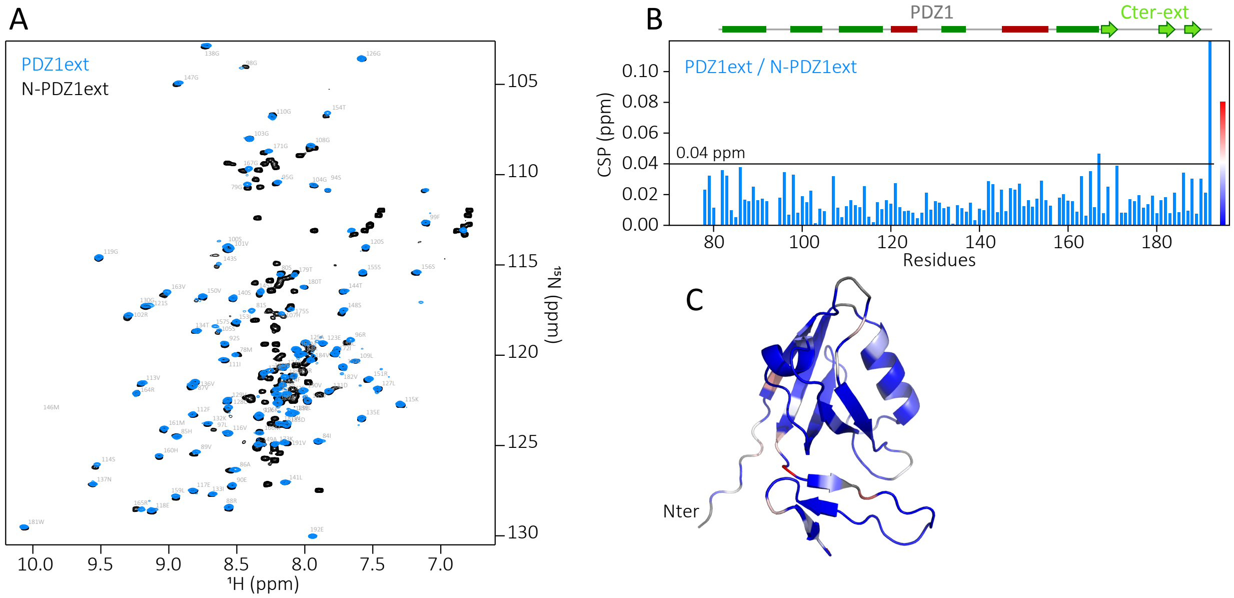

Supplementary 7: **(A)** Superimposition of PDZ1ext (black) and N-PDZ1ext (blue) ^{1}H-^{15}N HSQC spectra. **(B)** Chemical Shift Perturbations (CSP) measured for each residue of PDZ1ext when comparing PDZ1ext to N-PDZ1ext. Secondary structures are represented above the barplot, green for β strands of the PDZ core (from SSP), dark red for α helices of the PDZ core (from SSP) and light green arrows for β strands of the extension (from alphafold). **(C)** CSP are mapped on the alphafold model for PDZ1ext, from blue (no perturbation) to red (large perturbation). Residues depicted in grey have no associated chemical shift (missing peak in N-PDZ1ext).

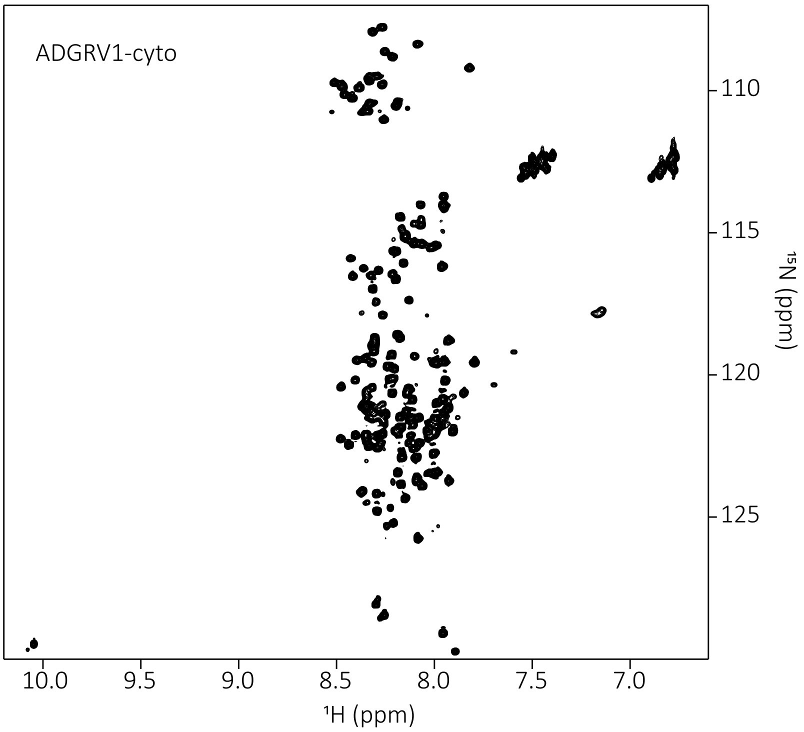

Supplementary 8: ^{1}H-^{15}N HSQC spectra of ADGRV1 whole cytoplasmic domain (152 residues).

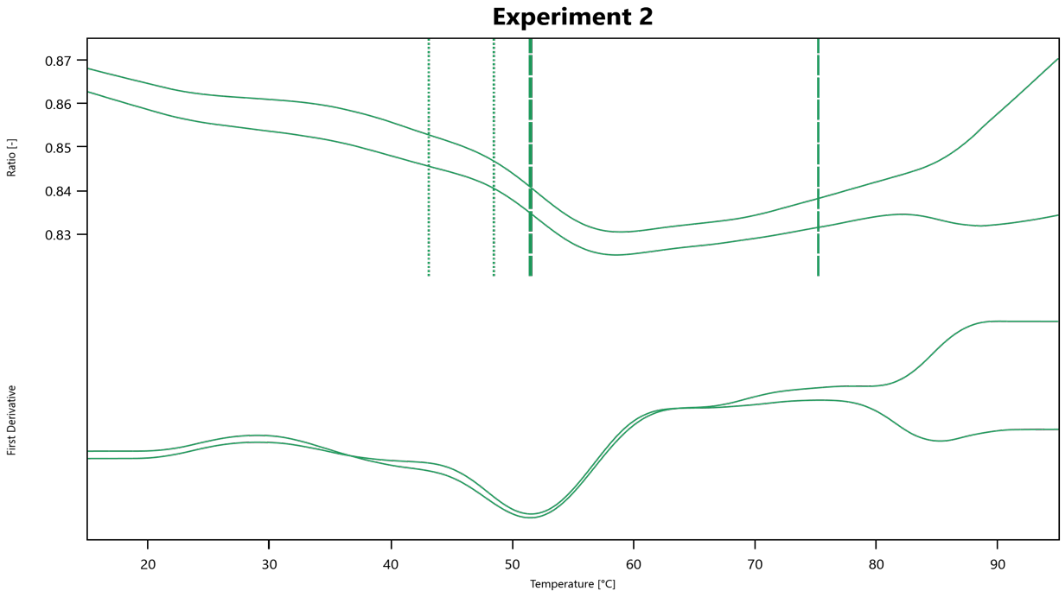

Supplementary 9: nanoDSF denaturation curves of PDZD7 PDZ2ext G228R, unbound and after addition of a peptide corresponding to the last 13 residues of ADGRV1. The transition of fluorescence emission ratio (330/350nm) starts below the temperature range of the spectrometer (15°C).

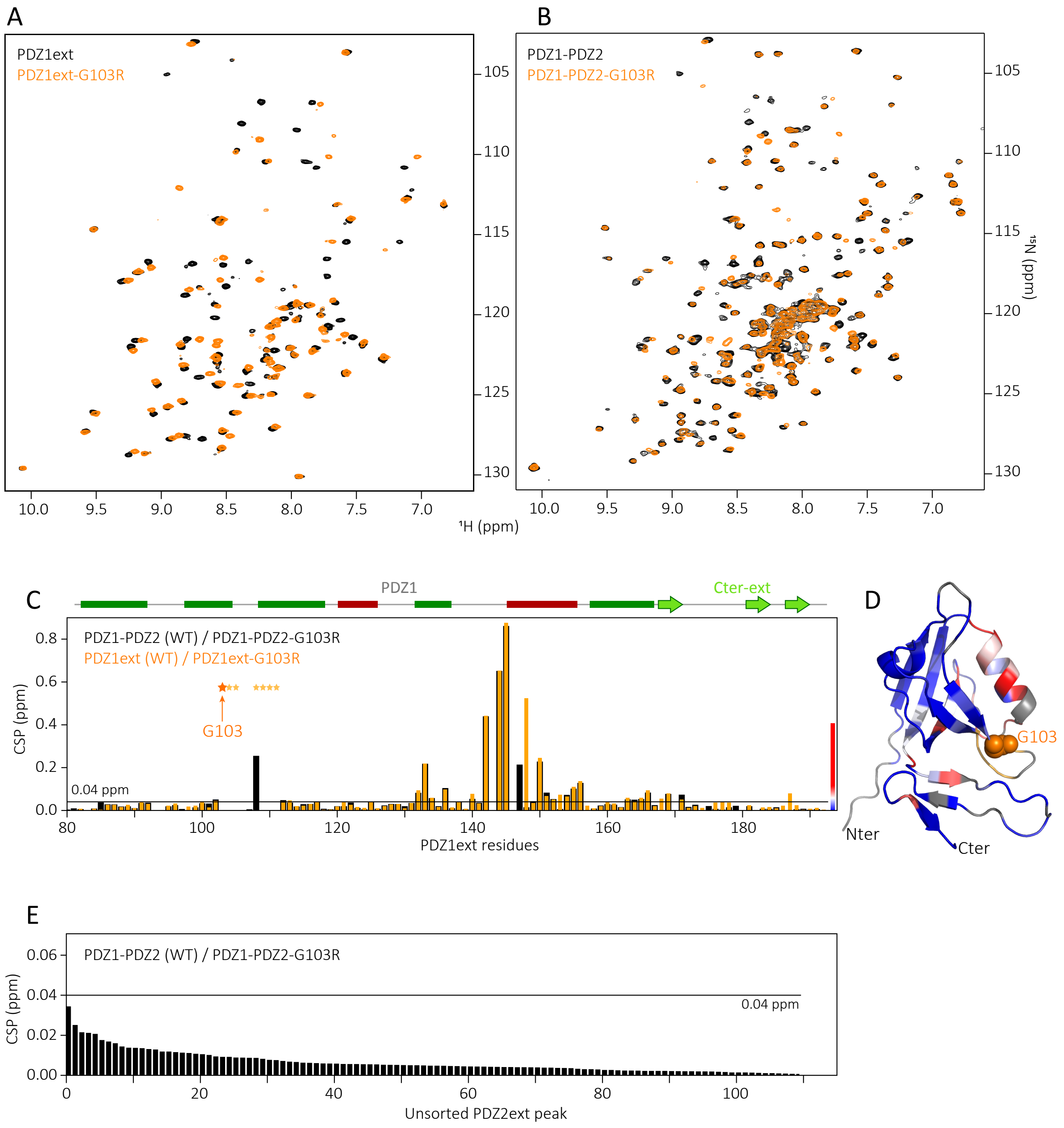

Supplementary 10: **(A)** Superimposition of PDZ1ext WT (black) and PDZ1ext G103R (orange) ^{1}H-^{15}N HSQC spectra. **(B)** Superimposition of P1P2 WT (black) and P1P2 G103R (orange) ^{1}H-^{15}N HSQC spectra. **(C)** Chemical Shift Perturbations (CSP) measured for each residue of PDZ1ext when comparing P1P2 WT to P1P2 G103R (black) and PDZ1ext WT to PDZ1ext G103R (orange). Secondary structures are represented above the barplot, green for β strands of the PDZ core (from SSP), dark red for α helices of the PDZ core (from SSP) and light green arrows for β strands of the extension (from alphafold). **(D)** Unsorted Chemical Shift Perturbations (CSP) measured for each residue of PDZ2ext when comparing P1P2 WT to P1P2 G103R.

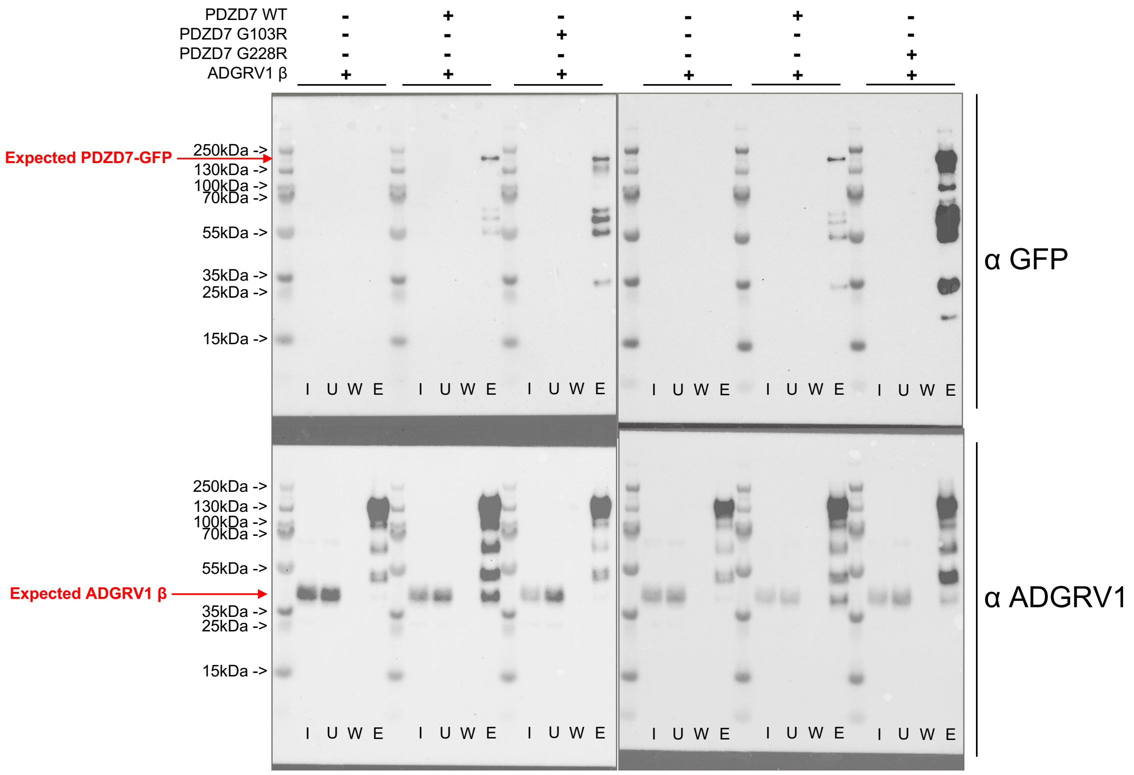

Supplementary 11: Full membranes corresponding to the figure 5 of the main text. Top membranes are read using an antibody directed against the GFP (fused to PDZD7). Bottom membranes are read using a custom antibody directed against the cytoplasmic domain of ADGRV1.
